# Crystal structure of (μ-hydrogen di­sulfato)-μ-oxido-bis­[(4,4′-di-*tert*-butyl-2,2′-bi­pyridine)­oxidovanadium(IV/V)] aceto­nitrile monosolvate

**DOI:** 10.1107/S2056989023009040

**Published:** 2023-10-19

**Authors:** Shintaro Kodama, Terushi Hashiguchi, Akihiro Nomoto

**Affiliations:** a Department of Applied Chemistry, Graduate School of Engineering, Osaka, Metropolitan University, 1-1 Gakuen-cho, Nakaku, Sakai, Osaka 599-8531, Japan; b Department of Applied Chemistry, Graduate School of Engineering, Osaka, Prefecture University, 1-1 Gakuen-cho, Nakaku, Sakai, Osaka 599-8531, Japan; Institute of Chemistry, Chinese Academy of Sciences

**Keywords:** crystal structure, vanadium, bi­pyridine, sulfate

## Abstract

The dinuclear oxidovanadium(IV/V) complex, [V_2_(HS_2_O_8_)O_3_(C_18_H_24_N_2_)_2_]·CH_3_CN or [V_2_O_2_(μ-O)(μ-H(SO_4_)_2_)(4,4′-^*t*^Bubpy)_2_]·CH_3_CN (4,4′-^*t*^Bubpy = 4,4′-di-*tert*-butyl-2,2′-bi­pyridine), has crystallographic *C*_2_ symmetry and exhibits a distorted octa­hedral geometry around the vanadium center, where the two 4,4′-^*t*^Bubpy ligands are nearly orthogonal to each other. The two vanadium ions are linked by an oxo anion and a unique protonated sulfate anion [H(SO_4_)_2_^3−^]. In the crystal, inter­molecular C—H⋯π and π–π inter­actions between the 4,4′-^*t*^Bubpy ligands are present, leading to a three-dimensional network.

## Abstract 2

The crystal structure of a dinuclear oxidovanadium(IV/V) complex [V_2_O_2_(μ-O)(μ-H(SO_4_)_2_)(4,4′-^*t*^Bubpy)_2_] aceto­nitrile monosolvate has been determined.

## 1. Chemical context

The sulfate anion (SO_4_^2–^) plays an important role as a ligand for transition-metal compounds, including polyoxometalates (Walsh *et al.*, 2016 ▸), metal sulfates (Natarajan & Mandal, 2008 ▸), and polynuclear complexes with organic ligands (Papatriantafyllopoulou *et al.*, 2009 ▸). Based on these compounds, a variety of catalysts (Wang *et al.*, 2021 ▸), magnetic materials (Gómez-García *et al.*, 2016 ▸), and metal–organic frameworks (Mi *et al.*, 2022 ▸) have been developed in recent years. A hydrogensulfate anion (HSO_4_^−^) is also found in transition-metal compounds, and HSO_4_^−^ more often acts as a counter-anion than as a ligand (Díaz-Torres & Alvarez, 2011 ▸). Thus, transition-metal complexes having an HSO_4_^−^ ligand are still limited in number. In sulfated metal oxide catalysts (*e.g.*, V_2_O_5_-based catalysts), however, a surface-protonated sulfate group is often proposed as a Brønsted acid site and affects the catalytic activity (Xie *et al.*, 2021 ▸). Hence, a transition-metal complex having a protonated sulfate anion as the ligand is expected to be an appropriate model compound to understand the active site of sulfated solid catalysts at the mol­ecular level. Herein, we report the crystal structure of a dinuclear oxidovanadium(IV/V) complex with 4,4′-di-*tert*-butyl-2,2′-bi­pyridine (4,4′-^*t*^Bubpy) ligands, the two vanadium ions of which are linked by an oxo anion and a unique protonated sulfate anion [H(SO_4_)_2_^3−^].

## 2. Structural commentary

**Figure 1:**
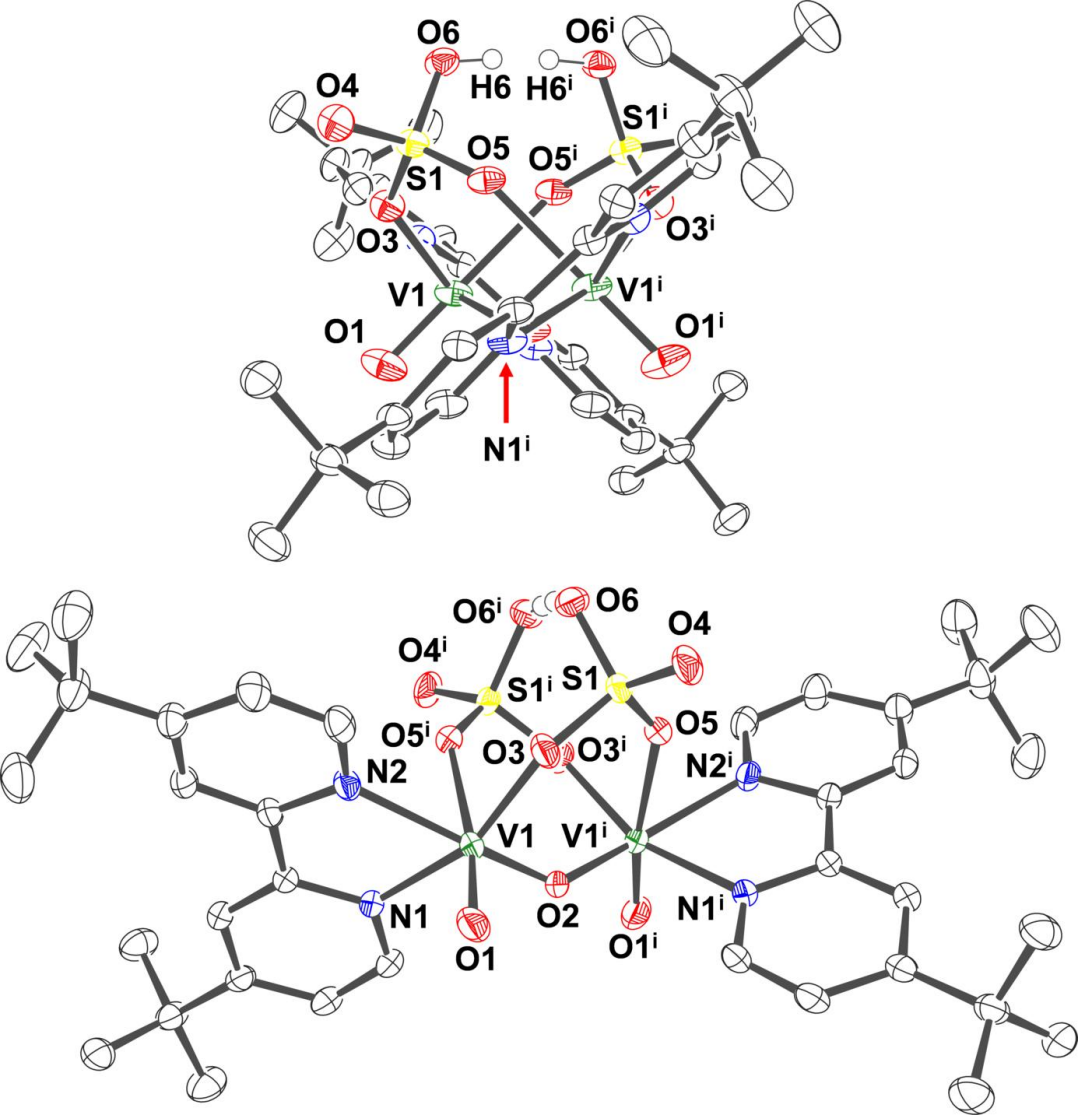
Crystal structure of [V_2_O_2_(μ-O)(μ-H(SO_4_)_2_)(4,4′-^*t*^Bubpy)_2_] (**V_2_**) with numbered atoms. Ellipsoids are shown at the 50% probability level. Side view (top) and front view (bottom). The hydrogen atoms of 4,4′-^*t*^Bubpy ligands are omitted for clarity. Symmetry code: (i) −*x* + 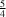 , y, −*z* + 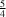 . The hydrogen atom of H(SO_4_)_2_^3–^ ligand is located at two positions (H6 and H6^i^) with 0.5 occupancy. Selected inter­atomic distances (Å): V1—O1 1.5932 (14), V1—O2 1.8268 (7), V1—O3 1.9692 (12), V1—O5^i^ 2.2827 (12), V1—N1 2.1077 (14), V1—N2 2.1399 (14), S1—O3 1.5098 (12), S1—O4 1.4391 (13), S1—O5 1.4654 (13), S1—O6 1.5066 (13), O6⋯O6^i^ 2.480 (2).

A single-crystal X-ray structure analysis revealed a novel dinuclear oxidovanadium(IV/V) complex [V_2_O_2_(μ-O)(μ-H(SO_4_)_2_)(4,4′-^*t*^Bubpy)_2_] (**V_2_**) with crystallographic *C*_2_ symmetry (Fig. 1 ▸). Complex **V_2_** exhibits a distorted octa­hedral geometry around the vanadium centre, where the two 4,4′-^*t*^Bubpy ligands are nearly orthogonal to each other [the dihedral angle between the coordination planes of N1–V1–N2 and N1^i^–V1^i^–N2^i^ is 86.48 (8)°]. The two vanadium ions are linked by bridging O^2–^ and H(SO_4_)_2_^3–^ ions. The lengths of the V=O_terminal_ [V1—O1; 1.5932 (14) Å], V—O_bridging_ [1.8268 (7)–2.2827 (12) Å], and V—N [2.1077 (14)–2.1399 (14) Å] bonds are within the expected values reported in the literature (Triantafillou *et al.*, 2004 ▸; Inoue *et al.*, 2018 ▸). For the S—O distances in the H(SO_4_)_2_^3–^ ion, the distances between S1 and O atoms (O3 and O5) attached to V atom are in the range of 1.4654 (13) to 1.5098 (12) Å, whereas the S=O_terminal_ [S1—O4; 1.4391 (13) Å] bond is substanti­ally shorter. Although, like the O4 atom, the O6 atom is not attached to the V atom, the S1—O6 distance [1.5066 (13) Å] is comparable in length to the S1—O3 distance. Therefore, the S1—O6 distance can be attributed to the S—OH bond (Leszczyński *et al.*, 2012 ▸). The hydrogen atom of the H(SO_4_)_2_^3–^ ligand is located with 0.5 occupancy at two positions (H6 and H6^i^) (Schindler & Wickleder, 2017 ▸) related by the *C*_2_ axis passing through the midpoint of O6⋯O6^i^ and the O2 atom. In addition, the O6⋯O6^i^ distance (2.48 Å) reflects the strong intra­molecular hydrogen-bond inter­action in the H(SO_4_)_2_^3–^ ligand (Cleland *et al.*, 1998 ▸). 
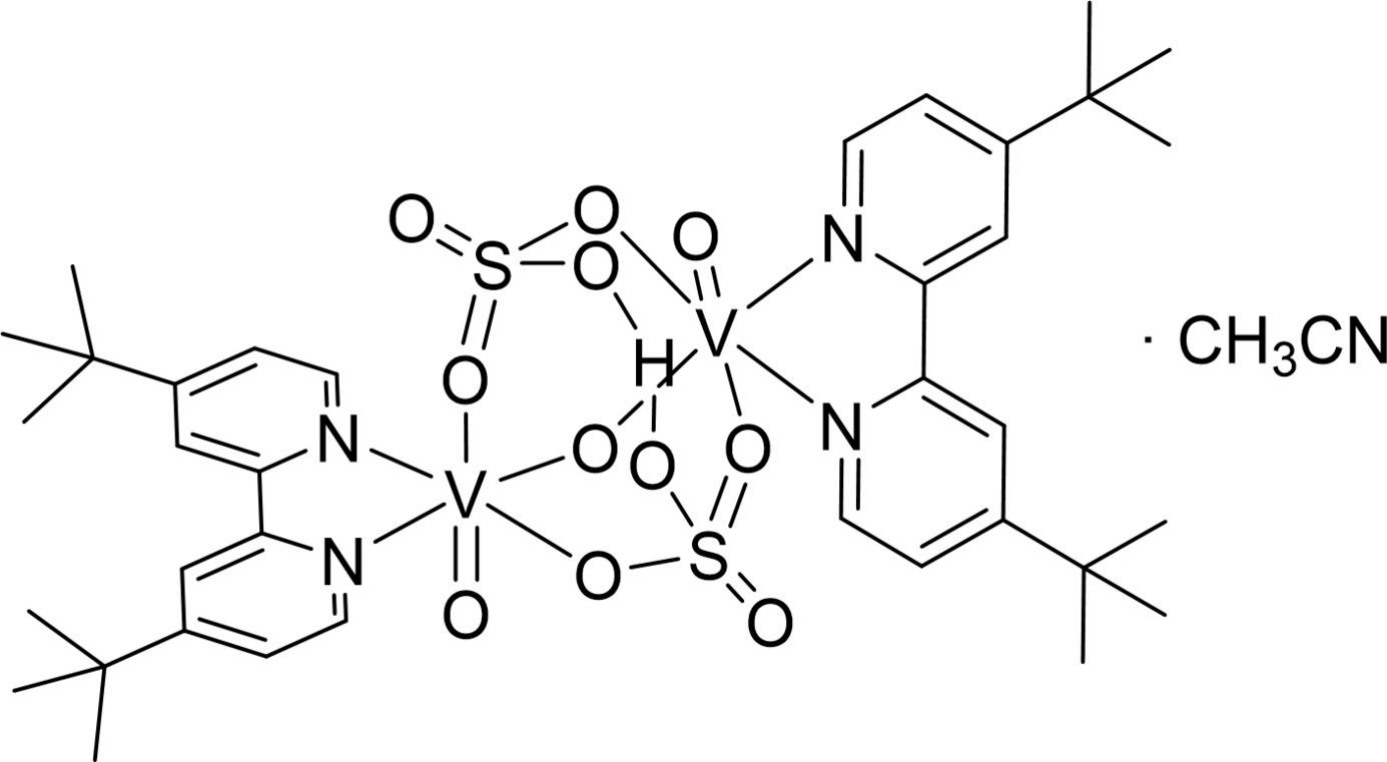


Bond-valence-sum calculations of complex **V_2_** (Table 1 ▸) suggest that the two V atoms (V1 and V1^i^) are in a mixed-valence state of V^4+^ and V^5+^. In addition, the UV-vis spectrum of **V_2_** in CH_3_CN shows two weak absorption bands at 553 nm (*ɛ* = 82 M^−1^ cm^−1^) and 669 nm (*ɛ* = 29 M^−1^ cm^−1^), which are considered to be the *d*–*d* bands of V^4+^ (Ballhausen & Gray, 1962 ▸; Waidmann *et al.*, 2009 ▸). To the best of our knowledge, the dinuclear structure of **V_2_** bearing the bridging H(SO_4_)_2_^3–^ ligand is unprecedented, although there are a few examples of vanadium complexes having the protonated sulfate anion (*e.g.*, HSO_4_^−^) as the ligand (Nilsson *et al.* 2009 ▸; Datta *et al.* 2015 ▸).

## 3. Supra­molecular features

**Figure 2:**
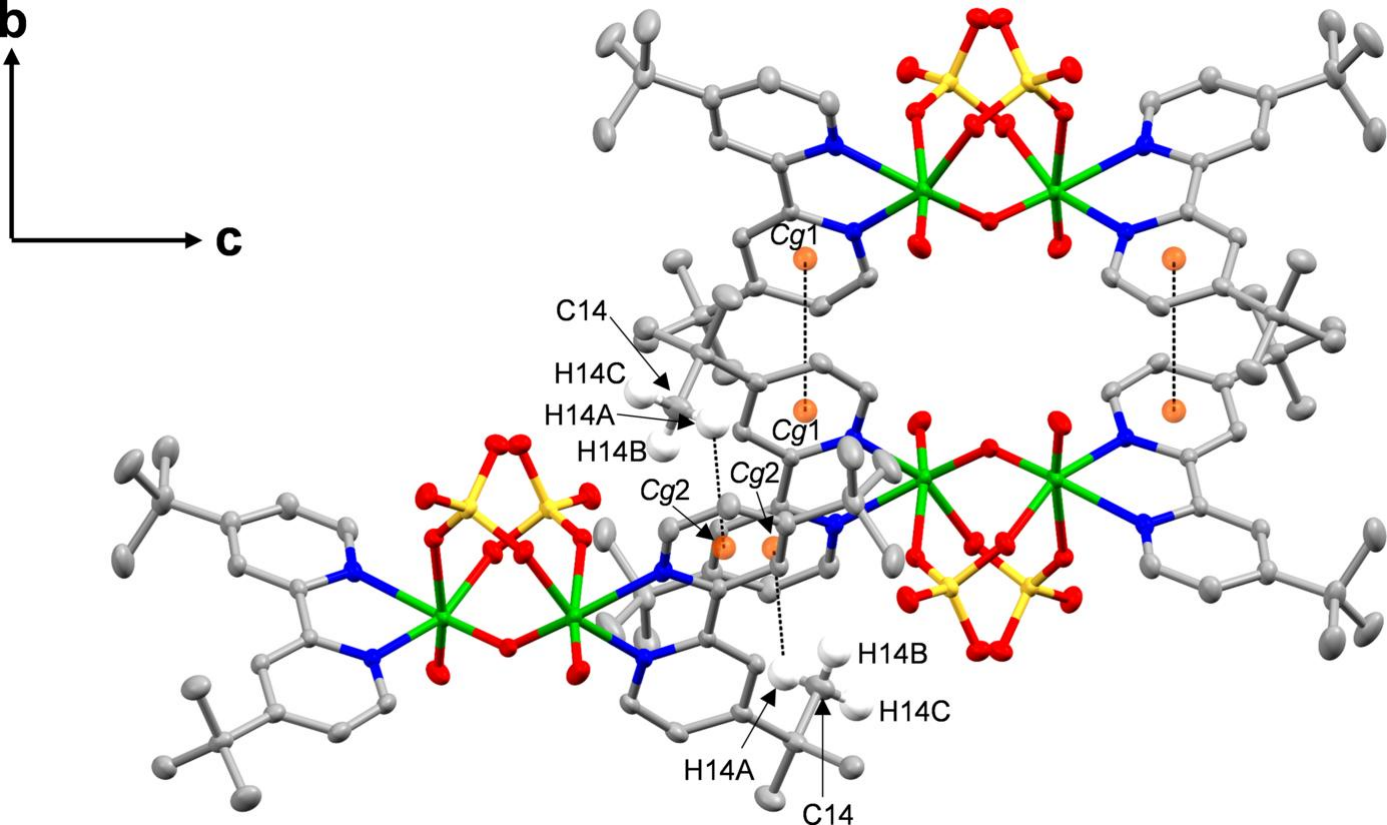
Details of the C—H⋯π inter­actions and the π–π inter­actions between the 4,4′-^*t*^Bubpy ligands of **V_2_**. *Cg*1 and *Cg*2 are the centroids of the N1/C1–C5 ring and the N2/C6–C10 ring, respectively. The hydrogen atoms of 4,4′-^*t*^Bubpy ligands except for H14*A*, H14*B*, and H14*C* are omitted for clarity.

**Figure 3:**
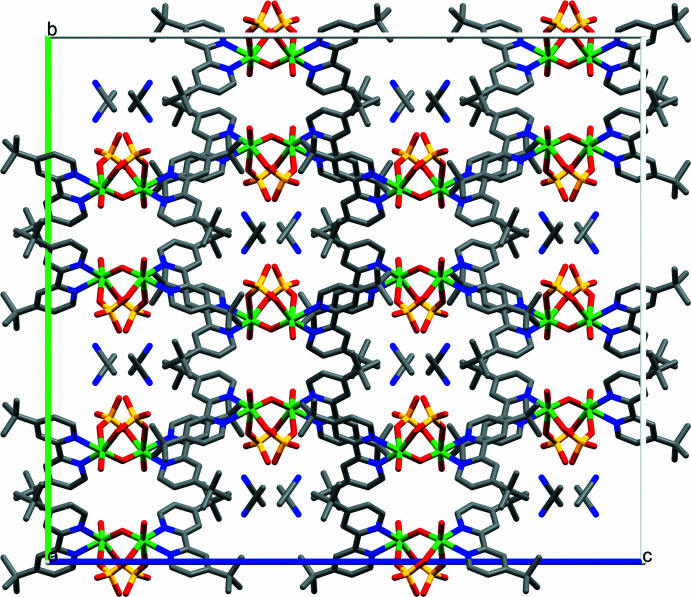
Crystal packing view of **V_2_** ·CH_3_CN along the *a* axis.

In the crystal of **V_2_**, inter­molecular C—H⋯π inter­actions (Karle *et al.*, 2007 ▸) between the H14*A* atom and the pyridine ring of the 4,4′-^*t*^Bubpy ligand (Table 2 ▸), along with inter­molecular π–π inter­actions (Janiak, 2000 ▸) between the pyridine rings of the 4,4′-^*t*^Bubpy ligands [*Cg*1⋯*Cg*1^i^ = 3.7222 (13) Å, inter­planar distance = 3.6034 (16) Å, slippage = 0.933 Å. *Cg*1 is the centroid of the N1/C1–C5 ring; symmetry code: (i) −*x* + 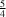 , −*y* + 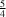 , *z*], are present (Fig. 2 ▸), forming a three-dimensional network (Fig. 3 ▸).

## 4. Synthesis and crystallization

To a solution of 4,4′-di-*tert*-butyl-2,2′-bipyridyl (4,4′-^*t*^Bubpy) (269.0 mg, 1.0 mmol) in EtOH (10 mL) was added a solution of VOSO_4_·5H_2_O (126.8 mg, 0.5 mmol) in EtOH (5.5 mL). After stirring for 2.5 h at 313 K, the solution was concentrated under reduced pressure, and the green precipitate was filtered using Et_2_O and dried to afford a green powder. Then, the powder was suspended in water, and an aqueous solution of sodium lauryl sulfate was added. After the mixture had been stirred overnight at ambient temperature, the supernatant liquid was separated from a dark-green oily precipitate by deca­ntation, and the precipitate was washed with water. The precipitate was dissolved in an EtOH–Et_2_O mixed solvent. After the color of the solution turned from green to orange, it was evaporated, and the precipitate was filtered using Et_2_O and dried to afford a yellowish brown solid, which was recrystallized from CH_3_CN and Et_2_O to give **V_2_** (30.8 mg, 13% based on V) as dark-brown crystals. Analysis calculated for C_36_H_49_N_4_O_11_S_2_V_2_·3H_2_O: C, 46.30; H, 5.94; N, 6.00. Found: C, 45.95; H, 5.82; N, 6.05.

## 5. Refinement

Crystal data, data collection and structure refinement details are summarized in Table 3 ▸. H atoms were positioned geom­etrically (C—H = 0.95–0.98 Å) and refined as riding with U_iso_(H) = 1.2*U*_eq_(C) or 1.5*U*_eq_(C_meth­yl_).

## Supplementary Material

Crystal structure: contains datablock(s) I. DOI: 10.1107/S2056989023009040/nx2001sup1.cif

Structure factors: contains datablock(s) I. DOI: 10.1107/S2056989023009040/nx2001Isup2.hkl

CCDC reference: 2301351

Additional supporting information:  crystallographic information; 3D view; checkCIF report

## Figures and Tables

**Table 1:** BVS calculations for vanadium atoms of [V_2_O_2_(μ-O)(μ-H(SO_4_)_2_)(4,4′-^*t*^Bubpy)_2_] (**V_2_**) BVS calculations were conducted using X-ray data of [V_2_O_2_(μ-O)(μ-H(SO_4_)_2_)(4,4′-^*t*^Bubpy)_2_]. Bond-valence parameters: V^IV^—O (1.784 Å), V^V^—O (1.803 Å), and V—N (1.86 Å) (Brese & O’Keeffe, 1991 ▸).

[V_2_O_2_(μ-O)(μ-H(SO_4_)_2_)(4,4′-^*t*^Bubpy)_2_]	V1
V(IV)	4.41
V(V)	4.59

**Table 2:** Hydrogen-bond geometry (Å, °) *Cg*2 is the centroid of the N2/C6–C10 ring.

*D*—H⋯*A*	*D*—H	H⋯*A*	*D*⋯*A*	*D*—H⋯*A*
C14—H14*A*⋯*Cg*2^i^	0.98	2.78	3.431 (2)	124

Symmetry code: (i) 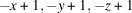 .

**Table 3:** Experimental details

Crystal data	
Chemical formula	[V_2_(HS_2_O_8_)O_3_(C_18_H_24_N_2_)_2_]·C_2_H_3_N
*M*_r_	920.84
Crystal system, space group	Orthorhombic, *F* *d* *d* *d*
Temperature (K)	110
*a*, *b*, *c* (Å)	13.0134 (2), 34.9495 (5), 39.4864 (6)
*V* (Å^3^)	17958.9 (5)
*Z*	16
Radiation type	Mo *K*α
μ (mm^−1^)	0.57
Crystal size (mm)	0.16 × 0.11 × 0.06

Data collection	
Diffractometer	Rigaku Saturn724+ (2×2 bin mode)
Absorption correction	Multi-scan (*CrysAlis PRO*; Rigaku OD, 2020 ▸)
*T*_min_, *T*_max_	0.815, 1.000
No. of measured, independent and observed [*I* > 2σ(*I*)] reflections	51528, 6582, 5675
*R*_int_	0.049
(sin θ/λ)_max_ (Å^−1^)	0.714

Refinement	
*R*[*F* ^2^ > 2σ(*F* ^2^)], *wR*(*F* ^2^), *S*	0.043, 0.110, 1.12
No. of reflections	6582
No. of parameters	287
No. of restraints	27
H-atom treatment	H atoms treated by a mixture of independent and constrained refinement
Δρ_max_, Δρ_min_ (e Å^−3^)	0.50, −0.34

Computer programs: *CrystalClear-SM Expert* (Rigaku, 2011 ▸), *OLEX2.solve* (Bourhis *et al.*, 2015 ▸), *SHELXL2018/3* (Sheldrick, 2015 ▸), and *OLEX2* (Dolomanov *et al.*, 2009 ▸).

## supplementary crystallographic information

### Crystal data

**Table:**

[V_2_(HS_2_O_8_)O_3_(C_18_H_24_N_2_)_2_]·C_2_H_3_N	*D*_x_ = 1.362 Mg m^−^^3^
*M**_r_* = 920.84	Mo *K*α radiation, λ = 0.71073 Å
Orthorhombic, *F**d**d**d*	Cell parameters from 30626 reflections
*a* = 13.0134 (2) Å	θ = 1.9–30.6°
*b* = 34.9495 (5) Å	µ = 0.57 mm^−^^1^
*c* = 39.4864 (6) Å	*T* = 110 K
*V* = 17958.9 (5) Å^3^	Block, clear dark brown
*Z* = 16	0.16 × 0.11 × 0.06 mm
*F*(000) = 7696	

### Data collection

**Table:**

Rigaku Saturn724+ (2x2 bin mode) diffractometer	6582 independent reflections
Radiation source: Rotating Anode	5675 reflections with *I* > 2σ(*I*)
Detector resolution: 28.5714 pixels mm^-1^	*R*_int_ = 0.049
profile data from ω–scans	θ_max_ = 30.5°, θ_min_ = 2.1°
Absorption correction: multi-scan (CrysAlisPro; Rigaku OD, 2020)	*h* = −18→18
*T*_min_ = 0.815, *T*_max_ = 1.000	*k* = −49→50
51528 measured reflections	*l* = −55→54

### Refinement

**Table:**

Refinement on *F*^2^	Primary atom site location: iterative
Least-squares matrix: full	Hydrogen site location: mixed
*R*[*F*^2^ > 2σ(*F*^2^)] = 0.043	H atoms treated by a mixture of independent and constrained refinement
*wR*(*F*^2^) = 0.110	*w* = 1/[σ^2^(*F*_o_^2^) + (0.0469*P*)^2^ + 46.648*P*] where *P* = (*F*_o_^2^ + 2*F*_c_^2^)/3
*S* = 1.12	(Δ/σ)_max_ = 0.003
6582 reflections	Δρ_max_ = 0.50 e Å^−^^3^
287 parameters	Δρ_min_ = −0.33 e Å^−^^3^
27 restraints	

### Special details

**Table:**

Geometry. All esds (except the esd in the dihedral angle between two l.s. planes) are estimated using the full covariance matrix. The cell esds are taken into account individually in the estimation of esds in distances, angles and torsion angles; correlations between esds in cell parameters are only used when they are defined by crystal symmetry. An approximate (isotropic) treatment of cell esds is used for estimating esds involving l.s. planes.

### Fractional atomic coordinates and isotropic or equivalent isotropic displacement parameters (Å^2^)

**Table:**

	*x*	*y*	*z*	*U*_iso_*/*U*_eq_	Occ. (<1)
V1	0.70713 (2)	0.54069 (2)	0.59096 (2)	0.02335 (8)	
S1	0.75306 (3)	0.47690 (2)	0.64590 (2)	0.02120 (10)	
O5	0.66572 (10)	0.49435 (3)	0.66352 (3)	0.0235 (2)	
O3	0.78535 (10)	0.50242 (4)	0.61695 (3)	0.0255 (3)	
O6	0.71873 (10)	0.43950 (3)	0.63066 (3)	0.0258 (3)	
O4	0.84010 (10)	0.46966 (4)	0.66746 (3)	0.0299 (3)	
O1	0.79258 (12)	0.57322 (4)	0.58859 (3)	0.0369 (3)	
O2	0.625000	0.55852 (5)	0.625000	0.0394 (5)	
N2	0.75091 (11)	0.51363 (4)	0.54454 (3)	0.0216 (3)	
N1	0.60817 (12)	0.56465 (4)	0.55441 (3)	0.0224 (3)	
C3	0.48418 (13)	0.59696 (5)	0.50377 (4)	0.0225 (3)	
C6	0.69971 (13)	0.52426 (5)	0.51604 (4)	0.0202 (3)	
C7	0.72724 (13)	0.51037 (5)	0.48439 (4)	0.0223 (3)	
H7	0.688735	0.517593	0.464972	0.027*	
C8	0.81169 (13)	0.48568 (5)	0.48092 (4)	0.0252 (3)	
C11	0.41640 (14)	0.61383 (5)	0.47598 (5)	0.0260 (3)	
C4	0.55602 (13)	0.56814 (4)	0.49641 (4)	0.0210 (3)	
H4	0.562535	0.558953	0.473872	0.025*	
C5	0.61741 (13)	0.55301 (4)	0.52172 (4)	0.0200 (3)	
C2	0.47668 (14)	0.60821 (5)	0.53749 (5)	0.0265 (3)	
H2	0.428897	0.627475	0.543821	0.032*	
C10	0.83025 (13)	0.48947 (5)	0.54148 (4)	0.0259 (3)	
H10	0.865230	0.481514	0.561393	0.031*	
C9	0.86340 (14)	0.47561 (5)	0.51049 (5)	0.0283 (4)	
H9	0.921500	0.459194	0.509397	0.034*	
C1	0.53844 (15)	0.59148 (5)	0.56180 (4)	0.0266 (3)	
H1	0.531067	0.599365	0.584695	0.032*	
C15	0.84388 (15)	0.47210 (6)	0.44572 (5)	0.0329 (4)	
C12	0.48286 (15)	0.62876 (5)	0.44658 (5)	0.0310 (4)	
H12A	0.524627	0.607783	0.437528	0.047*	
H12B	0.527993	0.649180	0.454815	0.047*	
H12C	0.438268	0.638807	0.428664	0.047*	
C13	0.34999 (16)	0.64692 (6)	0.48896 (6)	0.0375 (5)	
H13A	0.394311	0.666815	0.498583	0.056*	
H13B	0.302988	0.637465	0.506451	0.056*	
H13C	0.310203	0.657649	0.470150	0.056*	
C14	0.34585 (16)	0.58193 (6)	0.46305 (5)	0.0346 (4)	
H14A	0.299790	0.573763	0.481303	0.052*	
H14B	0.387562	0.560154	0.455598	0.052*	
H14C	0.305067	0.591466	0.443967	0.052*	
C18	0.93513 (18)	0.44437 (7)	0.44714 (6)	0.0434 (5)	
H18A	0.915526	0.421472	0.459928	0.065*	
H18B	0.993353	0.456879	0.458329	0.065*	
H18C	0.954806	0.437044	0.424081	0.065*	
C17	0.87660 (19)	0.50771 (7)	0.42503 (5)	0.0437 (5)	
H17A	0.929138	0.521948	0.437572	0.065*	
H17B	0.816724	0.524171	0.421258	0.065*	
H17C	0.904661	0.499545	0.403158	0.065*	
C16	0.75339 (19)	0.45218 (7)	0.42815 (6)	0.0467 (6)	
H16A	0.695186	0.469893	0.426686	0.070*	
H16B	0.733200	0.429568	0.441237	0.070*	
H16C	0.773966	0.444343	0.405306	0.070*	
N3	0.6842 (6)	0.5900 (2)	0.3294 (2)	0.102 (2)	0.5
C19	0.5721 (5)	0.64049 (17)	0.36076 (15)	0.0605 (14)	0.5
H19A	0.549690	0.660308	0.344825	0.091*	0.5
H19B	0.611988	0.652277	0.379037	0.091*	0.5
H19C	0.511740	0.627648	0.370330	0.091*	0.5
C20	0.6367 (5)	0.61212 (17)	0.34275 (17)	0.0616 (15)	0.5
H6	0.659 (3)	0.4376 (11)	0.6263 (12)	0.022 (11)*	0.5

### Atomic displacement parameters (Å^2^)

**Table:**

	*U*^11^	*U*^22^	*U*^33^	*U*^12^	*U*^13^	*U*^23^
V1	0.03845 (17)	0.01764 (13)	0.01395 (13)	−0.00275 (11)	−0.00195 (11)	0.00237 (10)
S1	0.0276 (2)	0.01950 (18)	0.01648 (18)	−0.00117 (15)	−0.00710 (15)	0.00280 (14)
O5	0.0351 (6)	0.0179 (5)	0.0175 (5)	0.0015 (5)	−0.0031 (5)	0.0012 (4)
O3	0.0312 (6)	0.0263 (6)	0.0188 (6)	−0.0014 (5)	−0.0056 (5)	0.0055 (5)
O6	0.0299 (7)	0.0197 (5)	0.0278 (6)	0.0026 (5)	−0.0082 (5)	−0.0035 (5)
O4	0.0337 (7)	0.0335 (7)	0.0225 (6)	−0.0012 (5)	−0.0126 (5)	0.0061 (5)
O1	0.0574 (9)	0.0273 (6)	0.0260 (7)	−0.0137 (6)	−0.0164 (6)	0.0075 (5)
O2	0.0866 (16)	0.0163 (8)	0.0154 (8)	0.000	−0.0162 (9)	0.000
N2	0.0258 (7)	0.0224 (6)	0.0165 (6)	−0.0021 (5)	−0.0045 (5)	0.0027 (5)
N1	0.0361 (8)	0.0169 (6)	0.0142 (6)	−0.0020 (5)	0.0000 (5)	−0.0007 (5)
C3	0.0267 (8)	0.0173 (7)	0.0236 (8)	−0.0009 (6)	−0.0029 (6)	−0.0032 (6)
C6	0.0248 (7)	0.0199 (7)	0.0160 (7)	−0.0009 (6)	−0.0030 (6)	0.0017 (6)
C7	0.0259 (8)	0.0238 (7)	0.0171 (7)	0.0025 (6)	−0.0047 (6)	−0.0001 (6)
C8	0.0259 (8)	0.0288 (8)	0.0207 (8)	0.0024 (7)	−0.0022 (6)	−0.0010 (6)
C11	0.0290 (8)	0.0202 (7)	0.0289 (8)	0.0042 (6)	−0.0069 (7)	−0.0053 (6)
C4	0.0267 (8)	0.0196 (7)	0.0167 (7)	0.0002 (6)	−0.0016 (6)	−0.0022 (6)
C5	0.0280 (8)	0.0163 (6)	0.0157 (7)	−0.0014 (6)	−0.0010 (6)	−0.0007 (5)
C2	0.0331 (9)	0.0202 (7)	0.0260 (8)	0.0017 (6)	0.0018 (7)	−0.0064 (6)
C10	0.0255 (8)	0.0306 (8)	0.0216 (8)	0.0004 (7)	−0.0069 (6)	0.0034 (7)
C9	0.0252 (8)	0.0342 (9)	0.0257 (8)	0.0061 (7)	−0.0041 (7)	0.0014 (7)
C1	0.0418 (10)	0.0195 (7)	0.0185 (7)	−0.0011 (7)	0.0019 (7)	−0.0046 (6)
C15	0.0333 (9)	0.0419 (10)	0.0234 (8)	0.0141 (8)	−0.0034 (7)	−0.0064 (8)
C12	0.0378 (10)	0.0261 (8)	0.0292 (9)	0.0026 (7)	−0.0079 (7)	0.0021 (7)
C13	0.0373 (10)	0.0317 (10)	0.0436 (11)	0.0128 (8)	−0.0062 (9)	−0.0083 (8)
C14	0.0348 (10)	0.0284 (9)	0.0407 (11)	−0.0005 (7)	−0.0120 (8)	−0.0066 (8)
C18	0.0416 (11)	0.0547 (13)	0.0337 (11)	0.0206 (10)	−0.0025 (9)	−0.0081 (10)
C17	0.0523 (13)	0.0544 (13)	0.0243 (9)	0.0141 (11)	0.0077 (9)	0.0033 (9)
C16	0.0444 (12)	0.0551 (14)	0.0404 (12)	0.0158 (10)	−0.0109 (10)	−0.0243 (11)
N3	0.088 (4)	0.100 (4)	0.117 (5)	−0.037 (3)	0.042 (4)	−0.029 (4)
C19	0.073 (3)	0.063 (3)	0.045 (3)	−0.037 (3)	0.003 (3)	−0.007 (2)
C20	0.053 (3)	0.065 (3)	0.067 (3)	−0.022 (3)	0.008 (3)	0.001 (2)

### Geometric parameters (Å, º)

**Table:**

V1—O5^i^	2.2827 (12)	C10—H10	0.9500
V1—O3	1.9692 (12)	C10—C9	1.385 (3)
V1—O1	1.5932 (14)	C9—H9	0.9500
V1—O2	1.8268 (7)	C1—H1	0.9500
V1—N2	2.1399 (14)	C15—C18	1.534 (3)
V1—N1	2.1077 (14)	C15—C17	1.549 (3)
S1—O5	1.4654 (13)	C15—C16	1.534 (3)
S1—O3	1.5098 (12)	C12—H12A	0.9800
S1—O6	1.5066 (13)	C12—H12B	0.9800
S1—O4	1.4391 (13)	C12—H12C	0.9800
O6—H6	0.80 (4)	C13—H13A	0.9800
N2—C6	1.360 (2)	C13—H13B	0.9800
N2—C10	1.339 (2)	C13—H13C	0.9800
N1—C5	1.359 (2)	C14—H14A	0.9800
N1—C1	1.337 (2)	C14—H14B	0.9800
C3—C11	1.526 (2)	C14—H14C	0.9800
C3—C4	1.405 (2)	C18—H18A	0.9800
C3—C2	1.392 (2)	C18—H18B	0.9800
C6—C7	1.388 (2)	C18—H18C	0.9800
C6—C5	1.486 (2)	C17—H17A	0.9800
C7—H7	0.9500	C17—H17B	0.9800
C7—C8	1.404 (2)	C17—H17C	0.9800
C8—C9	1.393 (2)	C16—H16A	0.9800
C8—C15	1.527 (2)	C16—H16B	0.9800
C11—C12	1.539 (3)	C16—H16C	0.9800
C11—C13	1.532 (2)	N3—C20	1.120 (9)
C11—C14	1.532 (2)	C19—H19A	0.9800
C4—H4	0.9500	C19—H19B	0.9800
C4—C5	1.384 (2)	C19—H19C	0.9800
C2—H2	0.9500	C19—C20	1.482 (8)
C2—C1	1.382 (3)		
			
O3—V1—O5^i^	85.44 (5)	N2—C10—H10	118.7
O3—V1—N2	90.49 (5)	N2—C10—C9	122.69 (15)
O3—V1—N1	160.47 (5)	C9—C10—H10	118.7
O1—V1—O5^i^	172.18 (6)	C8—C9—H9	119.9
O1—V1—O3	98.89 (7)	C10—C9—C8	120.11 (16)
O1—V1—O2	102.02 (7)	C10—C9—H9	119.9
O1—V1—N2	94.54 (7)	N1—C1—C2	122.68 (16)
O1—V1—N1	95.89 (6)	N1—C1—H1	118.7
O2—V1—O5^i^	83.64 (5)	C2—C1—H1	118.7
O2—V1—O3	98.66 (5)	C8—C15—C18	112.05 (16)
O2—V1—N2	159.52 (5)	C8—C15—C17	107.79 (16)
O2—V1—N1	90.62 (5)	C8—C15—C16	110.01 (17)
N2—V1—O5^i^	78.84 (5)	C18—C15—C17	108.31 (18)
N1—V1—O5^i^	78.52 (5)	C18—C15—C16	108.92 (18)
N1—V1—N2	75.63 (5)	C16—C15—C17	109.72 (18)
O5—S1—O3	109.23 (7)	C11—C12—H12A	109.5
O5—S1—O6	108.70 (8)	C11—C12—H12B	109.5
O6—S1—O3	107.02 (7)	C11—C12—H12C	109.5
O4—S1—O5	113.76 (8)	H12A—C12—H12B	109.5
O4—S1—O3	109.43 (8)	H12A—C12—H12C	109.5
O4—S1—O6	108.49 (8)	H12B—C12—H12C	109.5
S1—O5—V1^i^	143.30 (7)	C11—C13—H13A	109.5
S1—O3—V1	130.69 (8)	C11—C13—H13B	109.5
S1—O6—H6	117 (3)	C11—C13—H13C	109.5
V1—O2—V1^i^	140.11 (10)	H13A—C13—H13B	109.5
C6—N2—V1	117.24 (11)	H13A—C13—H13C	109.5
C10—N2—V1	124.13 (11)	H13B—C13—H13C	109.5
C10—N2—C6	118.41 (14)	C11—C14—H14A	109.5
C5—N1—V1	118.52 (11)	C11—C14—H14B	109.5
C1—N1—V1	122.95 (11)	C11—C14—H14C	109.5
C1—N1—C5	118.51 (15)	H14A—C14—H14B	109.5
C4—C3—C11	120.86 (15)	H14A—C14—H14C	109.5
C2—C3—C11	122.56 (15)	H14B—C14—H14C	109.5
C2—C3—C4	116.56 (15)	C15—C18—H18A	109.5
N2—C6—C7	121.55 (15)	C15—C18—H18B	109.5
N2—C6—C5	114.41 (14)	C15—C18—H18C	109.5
C7—C6—C5	123.97 (14)	H18A—C18—H18B	109.5
C6—C7—H7	119.8	H18A—C18—H18C	109.5
C6—C7—C8	120.33 (15)	H18B—C18—H18C	109.5
C8—C7—H7	119.8	C15—C17—H17A	109.5
C7—C8—C15	119.64 (15)	C15—C17—H17B	109.5
C9—C8—C7	116.86 (16)	C15—C17—H17C	109.5
C9—C8—C15	123.49 (16)	H17A—C17—H17B	109.5
C3—C11—C12	110.40 (15)	H17A—C17—H17C	109.5
C3—C11—C13	112.15 (15)	H17B—C17—H17C	109.5
C3—C11—C14	107.75 (14)	C15—C16—H16A	109.5
C13—C11—C12	108.28 (16)	C15—C16—H16B	109.5
C14—C11—C12	109.39 (15)	C15—C16—H16C	109.5
C14—C11—C13	108.84 (16)	H16A—C16—H16B	109.5
C3—C4—H4	119.7	H16A—C16—H16C	109.5
C5—C4—C3	120.58 (15)	H16B—C16—H16C	109.5
C5—C4—H4	119.7	H19A—C19—H19B	109.5
N1—C5—C6	114.19 (14)	H19A—C19—H19C	109.5
N1—C5—C4	121.35 (15)	H19B—C19—H19C	109.5
C4—C5—C6	124.43 (14)	C20—C19—H19A	109.5
C3—C2—H2	119.9	C20—C19—H19B	109.5
C1—C2—C3	120.28 (16)	C20—C19—H19C	109.5
C1—C2—H2	119.9	N3—C20—C19	178.4 (7)
			
V1—N2—C6—C7	175.43 (12)	C7—C6—C5—N1	−175.49 (16)
V1—N2—C6—C5	−1.51 (18)	C7—C6—C5—C4	2.7 (3)
V1—N2—C10—C9	−172.91 (14)	C7—C8—C9—C10	0.7 (3)
V1—N1—C5—C6	−0.61 (18)	C7—C8—C15—C18	−178.11 (18)
V1—N1—C5—C4	−178.82 (12)	C7—C8—C15—C17	62.8 (2)
V1—N1—C1—C2	177.27 (13)	C7—C8—C15—C16	−56.8 (2)
O5^i^—V1—O2—V1^i^	36.88 (3)	C11—C3—C4—C5	179.87 (15)
O5—S1—O3—V1	−24.59 (12)	C11—C3—C2—C1	178.68 (16)
O3—V1—O2—V1^i^	−47.50 (4)	C4—C3—C11—C12	−54.6 (2)
O3—S1—O5—V1^i^	14.21 (15)	C4—C3—C11—C13	−175.46 (16)
O6—S1—O5—V1^i^	−102.23 (13)	C4—C3—C11—C14	64.8 (2)
O6—S1—O3—V1	92.92 (11)	C4—C3—C2—C1	0.4 (3)
O4—S1—O5—V1^i^	136.79 (12)	C5—N1—C1—C2	−1.0 (3)
O4—S1—O3—V1	−149.72 (10)	C5—C6—C7—C8	174.54 (16)
O1—V1—O2—V1^i^	−148.59 (5)	C2—C3—C11—C12	127.22 (18)
N2—V1—O2—V1^i^	68.14 (17)	C2—C3—C11—C13	6.4 (2)
N2—C6—C7—C8	−2.1 (3)	C2—C3—C11—C14	−113.38 (19)
N2—C6—C5—N1	1.4 (2)	C2—C3—C4—C5	−1.9 (2)
N2—C6—C5—C4	179.52 (15)	C10—N2—C6—C7	0.7 (2)
N2—C10—C9—C8	−2.1 (3)	C10—N2—C6—C5	−176.23 (15)
N1—V1—O2—V1^i^	115.25 (4)	C9—C8—C15—C18	3.4 (3)
C3—C4—C5—N1	1.9 (2)	C9—C8—C15—C17	−115.7 (2)
C3—C4—C5—C6	−176.08 (15)	C9—C8—C15—C16	124.7 (2)
C3—C2—C1—N1	1.0 (3)	C1—N1—C5—C6	177.74 (15)
C6—N2—C10—C9	1.4 (3)	C1—N1—C5—C4	−0.5 (2)
C6—C7—C8—C9	1.3 (3)	C15—C8—C9—C10	179.22 (18)
C6—C7—C8—C15	−177.25 (17)		

Symmetry code: (i) −*x*+5/4, *y*, −*z*+5/4.

### Hydrogen-bond geometry (Å, º)

*Cg*2 is the centroid of the N2/C6–C10 ring.

**Table:**

*D*—H···*A*	*D*—H	H···*A*	*D*···*A*	*D*—H···*A*
C14—H14*A*···*Cg*2^ii^	0.98	2.78	3.431 (2)	124

Symmetry code: (ii) −*x*+1, −*y*+1, −*z*+1.
